# Identification of Hub Genes Associated With the Development of Stomach Adenocarcinoma by Integrated Bioinformatics Analysis

**DOI:** 10.3389/fonc.2022.844990

**Published:** 2022-05-24

**Authors:** Kehui Zhang, Jian Wang, YingYing Zhu, Xiaolin Liu, Jiacheng Li, Zhe Shi, Mengxing Cao, Yong Li

**Affiliations:** Shanghai Municipal Hospital of Traditional Chinese Medicine, Shanghai University of Traditional Chinese Medicine, Shanghai, China

**Keywords:** stomach adenocarcinoma, identification, TCGA, GEO, hub genes

## Abstract

**Objective:**

This study was conducted in order to gain a better understanding of the molecular mechanisms of stomach adenocarcinoma (STAD), which is necessary to predict the prognosis of STAD and develop novel gene therapy strategies.

**Methods:**

In this study, the gene expression profile of GSE118916 in the Gene Expression Omnibus (GEO) and The Cancer Genome Atlas Program (TCGA) was used to explore the differential co-expression genes of STAD and normal tissues.

**Results:**

A total of 407 STAD samples were collected, consisting of 375 from stomach adenocarcinoma tissues and 32 from normal tissues, as well as RNA-seq count data for 19,600 genes. Forty-two differentially expressed genes were screened by weighted gene co-expression network analysis (WGCNA) and differentially expressed gene analysis. According to the functional annotation analysis of the clusterProfiler R package, these genes were analyzed for GO function enrichment, digestion (biological process), tube bottom material membrane (cell component), and oxidoreductase activity (molecular function). The KEGG pathway was enriched in gastric acid secretion and chemical carcinogenesis. In addition, Cytoscape’s cytoHubba plug-in was used to identify seven hub genes (*EWSR1*, *ESR1*, *CLTC*, *PCMT1*, *TP53*, *HUWE1*, and *HDAC1*) in a protein–protein interaction (PPI) network consisting of 7 nodes and 11 edges. Compared with normal tissues, *CLTC* and *TP53* genes were upregulated in stomach adenocarcinoma (*P* < 0.05). *TP53* was expressed differently in stages II and IV, *EWSR1* was expressed differently in stages II and III, and *ESR1* was expressed differently in stages I–III. Among the seven hub genes, Kaplan–Meier analysis and TCGG showed that the expression levels of *HDAC1* and *CLTC* were significantly correlated with OS in patients with stomach adenocarcinoma (*P* < 0.05). GEPIA2 analysis showed that *ESR1* expression was closely correlated with OS and DFS in gastric adenocarcinoma (*P* < 0.05). Then, the expression of the genes and their correlations were revealed by the R2 Platform (http://r2.amc.nl). Finally, we collected 18 pairs of gastric mucosal tissues from normal people and cancer tissues from patients with stomach adenocarcinoma. The expression levels of the above seven hub genes and their relative protein expression were detected by RT-PCR and immunohistochemistry (IHC). The results showed that the gene and protein expression levels in stomach adenocarcinoma tissues were increased than those in the normal group.

**Conclusion:**

In summary, we believe that the identified hub genes were related to the occurrence of stomach adenocarcinoma, especially the expression of *ESR1*, *HDAC1*, and *CLTC* genes, which are related to the prognosis and overall survival of patients and may become the potential for the future diagnosis and treatment of STAD.

## Introduction

Stomach adenocarcinoma (STAD), the core of this primer, is one of the most common malignant tumors in the clinic, which seriously threatens human life and health and brings a huge economic burden to society. Stomach cancer is more common in developing countries, mainly in China (40%) (1, 2). Since the majority of patients are diagnosed at an advanced stage, the prognosis is relatively poor. The 5-year survival rate of patients with advanced STAD is usually <5% (3). The molecular mechanism of STAD has not yet been fully clarified, so it is imperative to look for approaches to predict prognosis and develop new target gene therapy strategies.

In recent years, bioinformatics analysis has been paid more and more attention as a research hotspot. Weighted gene co-expression network analysis (WGCNA) is a method to understand the relationship between gene function and phenotype from genome-wide expression (4). It is applied to screen the co-expression modules of genes that are highly related to clinical diseases, identify gene modules related to clinical features, and finally find the key genes in the disease for further verification. In addition, the traditional biomarkers are still used clinically, and the lack of effective biomarkers for early STAD detection limits the treatment of the disease (5, 6). Differential gene expression analysis is a particular technique, which supplies an approach for studying the molecular mechanism of genome regulation and discovering the difference between the two groups of gene expression (7). These differentially expressed genes may be potential biomarkers to reveal diseases. As a result, the combination of WGCNA and differential gene expression analysis could better identify the core genes which may be used as potential biomarkers for STAD.

In the present study, disease-related mRNA data were obtained from The Cancer Genome Atlas Stomach Adenocarcinoma (TCGA-STAD) and Gene Expression Omnibus (GEO) databases, and differential co-expression genes were finally derived through WGCNA analysis and differential gene expression analysis. Next, we discussed the progress of STAD through Gene Ontology (GO) enrichment analysis, KEGG signal pathway analysis, and protein–protein interaction (PPI) analysis. Meanwhile, we applied online tools and clinical data in the database to verify survival analysis. We also verified differences in the expression of core genes between normal and stomach adenocarcinoma tissues. This study provides a potential basis for exploring the prognosis of STAD and target gene therapy by analyzing the differentially co-expressed genes of STAD.

## Method

**Figure 1:**
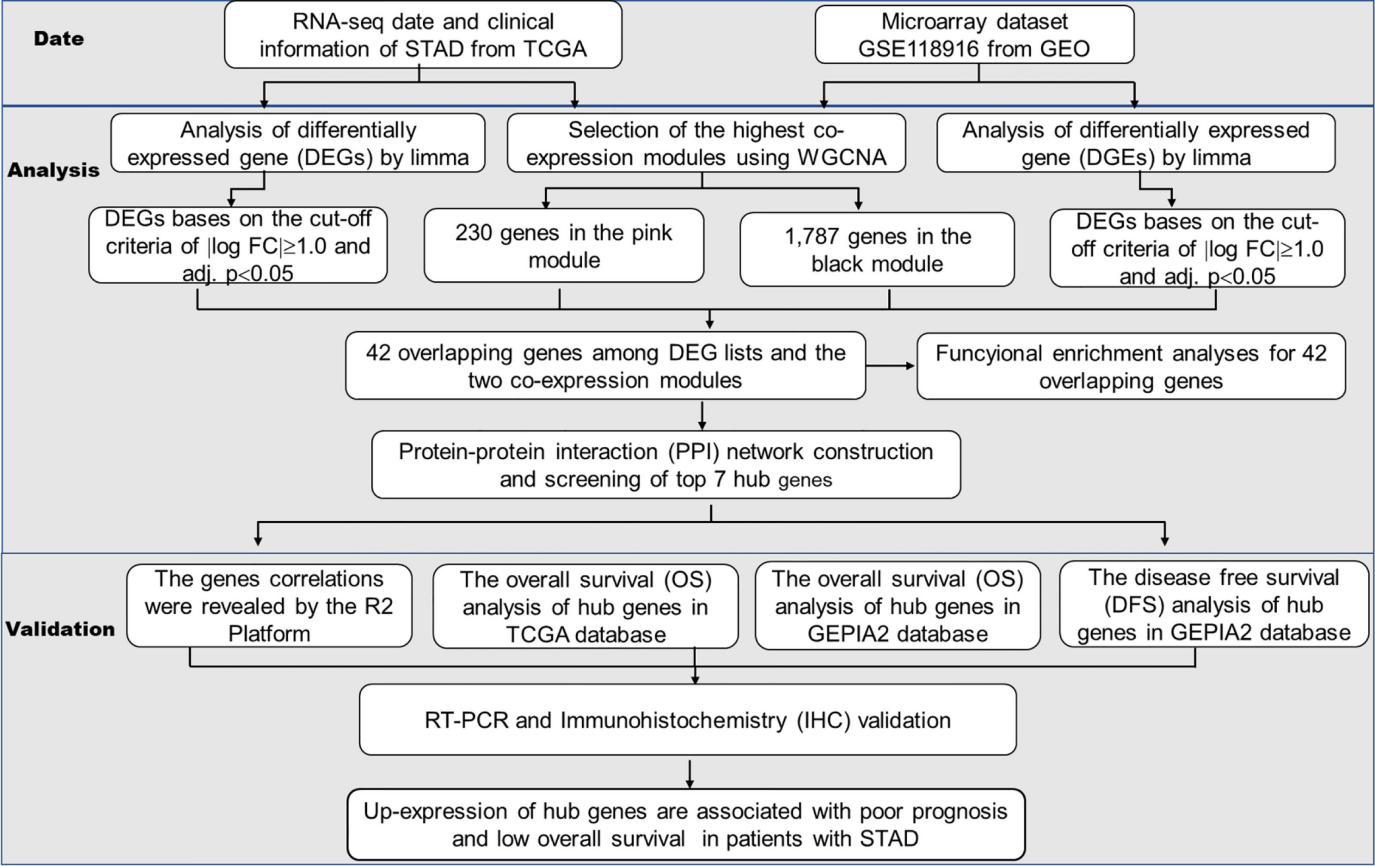
The workflow of the analysis of the hub gene extraction curation pipeline.

The analysis of the hub gene extraction management process is shown in **Figure 1**. We will discuss the specific steps in the subsequent sections.

### Database Extracted From the TCGA and GEO

The TCGA (https://portal.gdc.cancer.gov/) and GEO (https://www.ncbi.nlm.nih.gov/gds) were used to download the gene expression profile of STAD. All data of STAD including clinical information are available for free download through the R package TCGAbiolinks. In total, 407 STAD samples, which include 375 stomach adenocarcinoma and 32 normal tissues, and RNA-seq count data on 19,600 genes were collected. According to the recommendations of the edgeR package guide, genes with low reads were usually meaningless for analysis. Therefore, we kept genes with cpm (counts per million) ≥1. Furthermore, another normalized expression profile of STAD gene GSE118916 from GEO was gained by the R package GEOquery. GSE118916 including 15 cases of patients with STAD tumor specimen pairing and 15 cases of normal tissue was analyzed by using the GPL15207 platform Affymetrix Human Gene Expression Array. Probes were converted to gene markers according to the annotations provided by the producers, and duplicated microprobes of identical genes were removed by defining the midpoint expression value of all related microprobes. Therefore, 18,835 genes were chosen for the succeeding work.

### Recognition of Crucial Co-Expression Modules Based on WGCNA

Gene co-expression networks promoted the selection of genes, which can be used to identify potential biomarkers and drug targets. With the help of the WGCNA package, we built the gene expression profiles of TCGA-STAD and GSE118916. With the help of the function pickSoftThreshold, we set soft power *β* = 3 and 20 to create a scaleless network. Then, the adjacency matrix was created by formula and was transformed into a topological overlap matrix (TOM) as well as the corresponding dissimilarity (1-TOM). After that, similar expression genes were divided into different co-expression modules by constructing the hierarchical clustering tree of the 1-TOM matrix. Module characteristic associations and clinical characteristic information between modules were calculated based on previous studies so that further identification of functional modules in the co-expression network could be conducted. Consequently, modules related to clinical characteristic information and chosen for subsequent analysis were those with high correlation coefficients.

### Differential Expression Analysis and Comparison of Modules of Interest

The solution for RNA sequencing and differential expression analysis of microarray data was provided by the R package limma which was applied to screen differentially expressed genes (DEGs) in the TCGA-STAD and GSE118916 datasets to find the DEGs between STAD and normal tissues, respectively. The false discovery rate (FDR) was controlled by *P*-value which was adjusted by the Benjamini–Hochberg method. Genes with the cutoff criteria of |logFC| ≥1.0 and adj. *P <*0.05 were regarded as DEGs. The DEGs of the TCGA-STAD and GSE118916 datasets were envisaged as heat maps and volcano plots by the R package ggplot2. After that, the coincident genes between DEGs and the co-expression genes that were screened from the co-expression network were used to verify potential prognostic genes, which were shown in a Venn diagram through the R package VennDiagram.

### PPI Construction and Hub Gene Screening

In this research, we used STRING (a tool for online searching of interacting genes) to predict PPI and constructed a PPI network. Based on the STRING database, genes with scores ≥0.4 were screened to construct a network and displayed visually through Cytoscape (V3.7.2). In co-expression networks, the most effective method to combine the central nodes was the maximum clique centrality (MCC) algorithm which was calculated by Cytoscape’s plug-in (cytoHubba). In this research, the genes with the highest MCC values were extracted as hub genes.

### Expression Pattern and Prognostic Value of the Hub Gene

With the purpose of asserting the dependability of the hub gene, we assert the expression pattern of the hub gene in normal tissues and stomach adenocarcinoma tissues. The expression degree of the hub gene between the tumor and normal tissue was represented by a box diagram. Based on the clinical information from the TCGA-SATD, the Kaplan–Meier univariate survival analysis was performed by the survival package to find the relationship between the overall survival (OS) of the patient and the hub gene. We used the online tool GEPIA2 to determine the relationship between hub gene expression and disease-free survival (DFS) in STAD. Patients selected for this study completed the follow-up period and were separated into two groups based on the median expression value of the hub gene. Log-rank *P <*0.05 was considered statistically significant.

### The Gene Expression Correlations Revealed on the R2 Platform

The gene expression correlations were revealed by the R2: Genomics Analysis and Visualization Platform (http://r2.amc.nl).

### RNA Isolation and Real-Time Polymerase Chain Reaction

Total RNA was extracted using the TRIzol reagent (Invitrogen, USA). PrimeScript^®^ RT reagent kit (TaKaRa, Japan) and SYBR Premix Ex Taq (TaKaRa, Japan) were used for reverse transcription and quantitative real-time polymerase chain reaction (RT-PCR), respectively. The primers were synthesized by Shanghai Shenggong Biology Co., Ltd. The expression levels of genes relative to β-actin mRNA levels in each sample were calculated according to the 2−ΔΔCt method. The primer sequences are shown below: *EWSR1* forward, 5′-AGAACTTCGCCTGGAGAACA-3′ and reverse, 5′-GCCACCTCTGAACATTCCAC-3′; *ESR1* forward, 5′-CAAGCC CGCTCATGATCAAA-3′ and reverse, 5′-TCAAATCCACAAAGCCTGGC-3′; *CLTC* forward, 5′-TATCCGTCGGTTCCAGAGTG-3′ and reverse, 5′-AAGTGCCAATGTAGGGTCCA-3′; *PCMT1* forward, 5′-GTTCTGTAC CTGCTCCGAGT-3′ and reverse, 5′-ATTTTGCATAGTGGGAGCGG-3′; *HUWE1* forward, 5′-GGGAATCCTGGTGTGACTGA-3′ and reverse, 5′-CTGGATGAAGGTCACAGGGT-3′; *HDAC1* forward, 5′-ACCAAGTACCAC AGCGATGA-3′ and reverse, 5′-CCTCGGACTTCTTTGCATGG-3′; and *TP53* forward, 5′-GCCCCTCCTCAGCATCTTAT-3′ and reverse, 5′-AAAGCTGTTCCGTCCCAGTA-3′.

### Protein Expression Was Validated by Immunohistochemistry

Twenty tissue samples were examined: 10 from stomach adenocarcinoma tissues and the others from normal tissues. In brief, the tissue specimens were dewaxed and treated with methanol that contained 3% hydrogen peroxide to deactivate the endogenous peroxidase. Subsequently, the tissue specimens were treated with the primary antibody of GMFG at 4°C overnight and then treated with the secondary antibody (HRP polymer) for 30 min. Furthermore, diaminobenzoquinone (DAB) was used for the next step.

### Statistical Analyses

Statistical analyses were carried out using GraphPad Prism 8.0 software (GraphPad Software, Inc., La Jolla, CA, USA). Multiple comparisons were performed with the ANOVA test.

## Results

### Construction of Weighted Gene Co-Expression Modules

**Figure 2:**
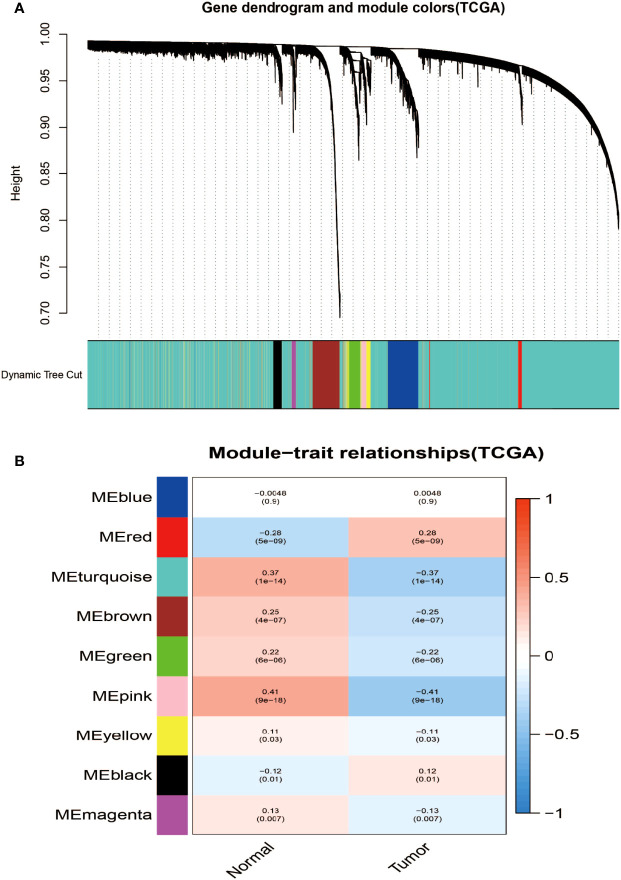
Identification of modules associated with the clinical information in the TCGA-STAD dataset. **(A)** The cluster dendrogram of co-expression network modules was ordered by a hierarchical clustering of genes based on the 1-TOM matrix. Each module was assigned different colors. **(B)** Module–trait relationships. Each row corresponds to a color module and each column corresponds to a clinical trait (cancer and normal). Each cell contains the corresponding correlation and *P*-value.

**Figure 3:**
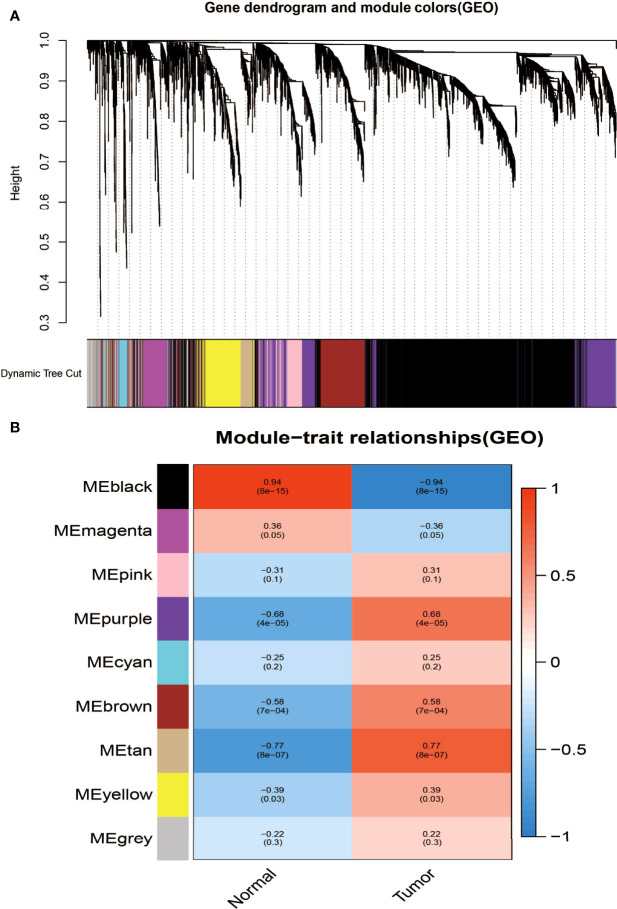
Identification of modules associated with clinical information in the GSE118916 dataset. **(A)** The cluster dendrogram of co-expression network modules was ordered by a hierarchical clustering of genes based on the 1-TOM matrix. Each module was assigned different colors. **(B)** Module–trait relationships. Each row corresponds to a color module and each column correlates to a clinical trait (cancer and normal). Each cell contains the corresponding correlation and *P*-value.

Aiming at finding the functional collection of STAD patients, the WGCNA software package was used for establishing a gene co-expression network which was gained from the TCGA-STAD and GSE118916 datasets. There are nine modules in TCGA-STAD, each of which is assigned a color (**Figure 2A**). A total of nine modules in TCGA-STAD (**Figure 2A**) and nine modules in GSE118916 (**Figure 3A**) were identified in our research since each one of them was identified as one different color. We then produced heat maps of the module–feature relationship to assess the connection of each module with two clinical manifestations. The final outcomes of the model–character relationship are shown in **Figures 2B**, **3B**, indicating that the pink module in TCGA-STAD and the black module in GSE118916 have the strongest correlation with normal tissues (pink module: *R* = 0.41, *P* = 9E−18; black module: *R* = 0.94, *P* = 8E−15).

The final outcomes of the model–character relationship are shown in **Figures 2B**, **3B**, indicating that the pink module in TCGA-STAD and the black module in GSE118916 have the strongest correlation with normal tissues (pink module: *r* = 0.41, *P* = 9E−18; black module: *R* = 0.94, *P* = 8E−15).

### Identification of Genes Between the DEG Lists and Co-Expression Modules

**Figure 4:**
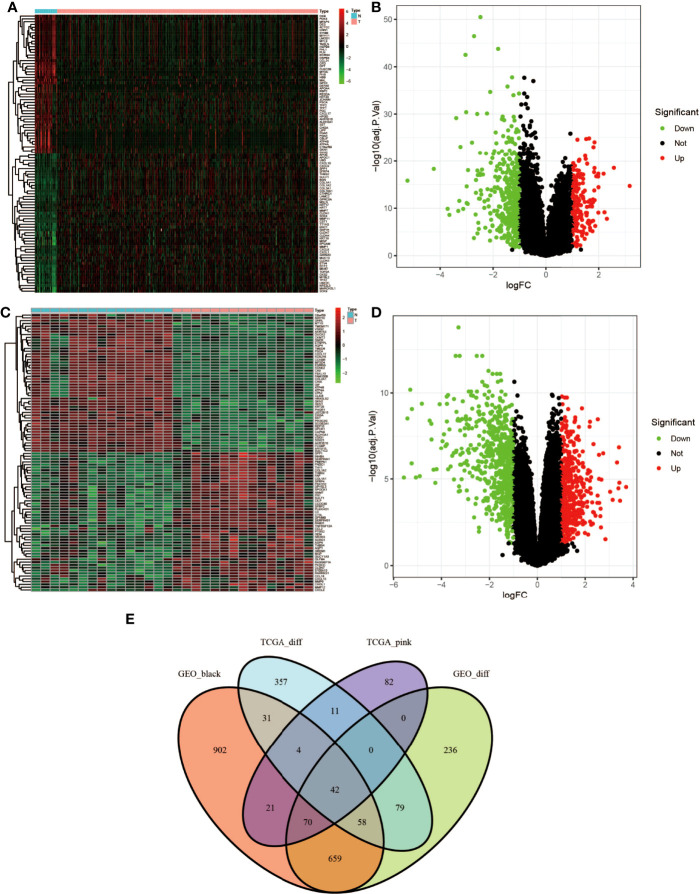
Identification of differentially expressed genes (DEGs) of the TCGA and GSE118916 datasets of STAD with the cutoff criteria of |logFC| ≥1.0 and adj. *P <*0.05. **(A, B)** Volcano plot of DEGs in the TCGA dataset. **(C, D)** Volcano plot of DEGs in the GSE118916 dataset. **(E)** The Venn diagram of genes among DEGs and co-expression module. In total, there were 42 overlapping genes in the intersection of DEGs and two co-expression modules.

According to |log FC| ≥1.0 and adj. truncation standard *P <*0.05, the limma software package found that there were 582 DEGs in the TCGA dataset (**Figures 4A, B**, red represents upregulated genes, and green represents downregulated genes) and 1,144 DEGs in the GSE118916 dataset (**Figures 4C, D**, red represents upregulated genes, green represents downregulated genes) in the tumor tissue expression disorder. As shown in **Figure 4E**, 230 and 1,787 co-expressed genes were found in the pink module of the TCGA dataset and in the black module of GSE118916, respectively. After crossing the four module genes (the TCGA differential gene, the GEO differential gene, the TCGA pink modular gene, and the GEO black modular gene), a total of 42 overlapping genes were extracted to verify the genes of co-expression modules (**Figure 4E**).

### Functional Enrichment Analyses for the 42 Genes

**Figure 5:**
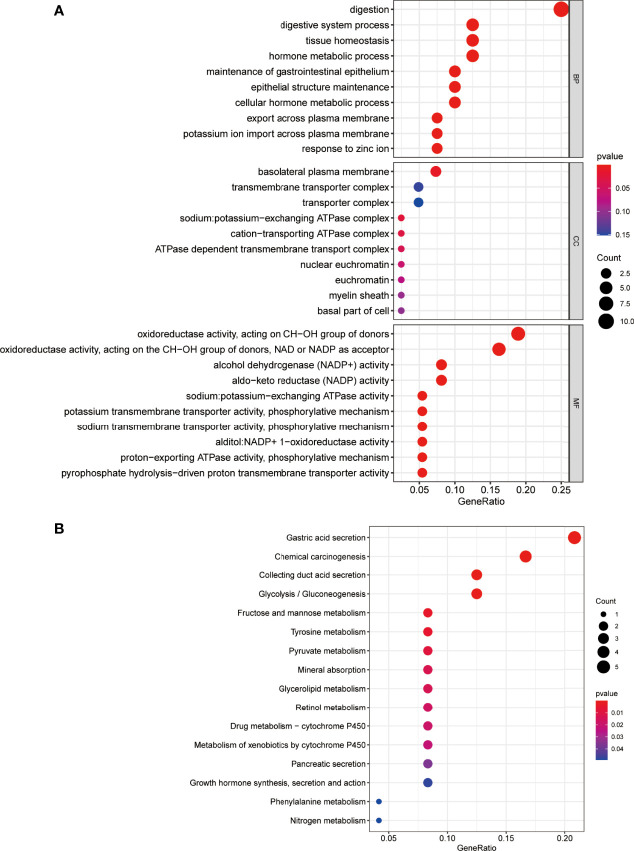
Enrichment analysis for the genes. **(A)** The bubble chart of GO enrichment analysis. **(B)** KEGG signal pathway analysis. The color represents the adjusted *P*-values (BH), and the size of the spots represents the gene number.

GO which covers three aspects of biology is widely used in the field of bioinformatics. clusterProfiler (v:3.14.3), org.Hs.eg.db (v:3.10.0), and enrichplot (v:1.6.1) in the R language were used to pair 42 of the final PPI subnets. Co-expressed genes were enriched, *P*-value <0.05 was selected as the screening criterion, and the first 20 genes were selected to draw the bubble chart of GO enrichment analysis (**Figure 5A**) and KEGG signal pathway analysis (**Figure 5B**) by the R language ggplot2 (v:3.2.1) package. In GO enrichment analysis, the biological process mainly involves digestion, the digestive system process, tissue homeostasis, hormone metabolism, and maintenance of gastrointestinal epithelium enrichment. The analysis of cell components showed that these genes were mainly engaged in the basolateral plasma membrane, transmembrane transporter complex, transporter complex, sodium:potassium-exchanging ATPase, ATPase-dependent transmembrane transport complex, etc. In addition, the molecular function analysis showed that these 42 genes were related to oxidoreductase activity, alcohol dehydrogenase (NADP^+^) activity, aldehyde ketone reductase (NADP) activity, sodium–potassium exchange ATPase activity, etc. KEGG signaling pathway analysis mainly involves gastric acid secretion, chemical carcinogenesis, collecting duct acid secretion, glycolysis/gluconeogenesis, fructose and mannose metabolism, tyrosine metabolism, and other signal pathways.

### PPI Network Construction and Hub Gene Identification of Co-Expressed Genes

**Figure 6:**
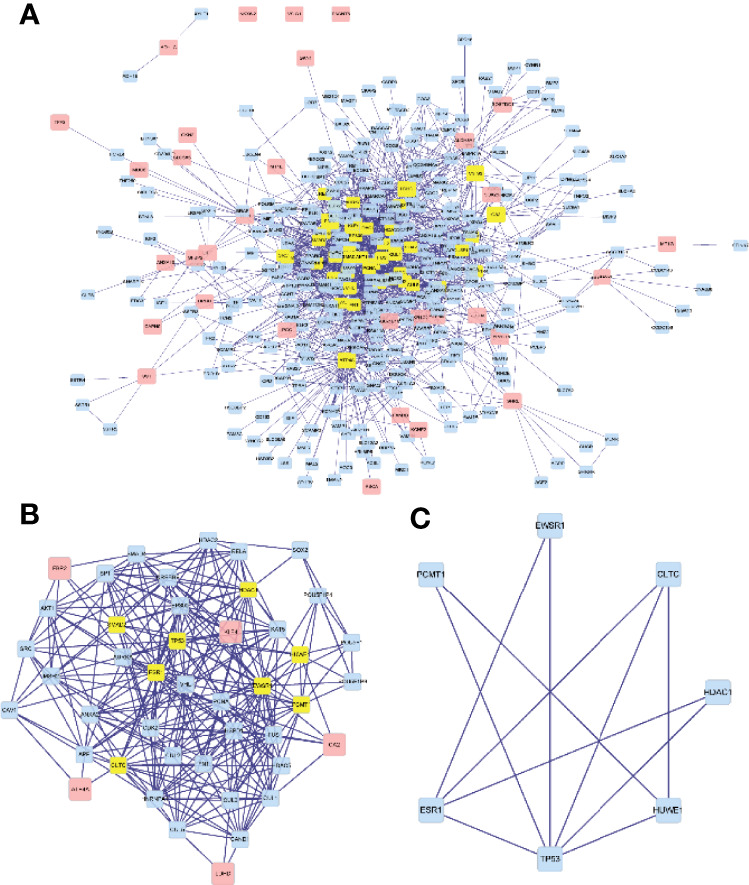
Visualization of the protein–protein interaction (PPI) network and the candidate hub genes. **(A)** A protein interaction network. **(B)** Identification of the hub genes from the PPI network in which the screening criterion is degree (DC) >20. **(C)** The final PPI subnetwork was screened according to BC >60.

We used the “BioGENET” (V:3.0.0) plug-in in Cytoscape (V:3.7.2) to construct a protein interaction network commonly used by the target genes (**Figure 6A**). The “Cytonca” (V:2.7.6) plug-in in Cytoscape (V:3.7.2) was used to analyze the PPI network topology and identify core genes in the network. The screening criteria is degree centrality (DC) >20 to get a new PPI network (**Figure 6B**), and then the betweenness centrality (BC) >60 was used to be the final PPI subnetwork (**Figure 6C**), which includes 7 nodes, 11 side, and 7 core genes. The seven hub genes are from high to low sorting as shown in **Table 1**, according to BC.

**Table 1:** Betweenness (BC) from high to low ranking of the seven hub genes.

Hub genes	Betweenness
EWSR1	161.13962
ESR1	104.66874
CLTC	92.86817
PCMT1	78.69666
TP53	76.55649
HUWE1	63.768787
HDAC1	61.19294

### Hub Gene Expression Pattern, Correlation, Prognostic Value, and Verification of Protein Expression

**Figure 7:**
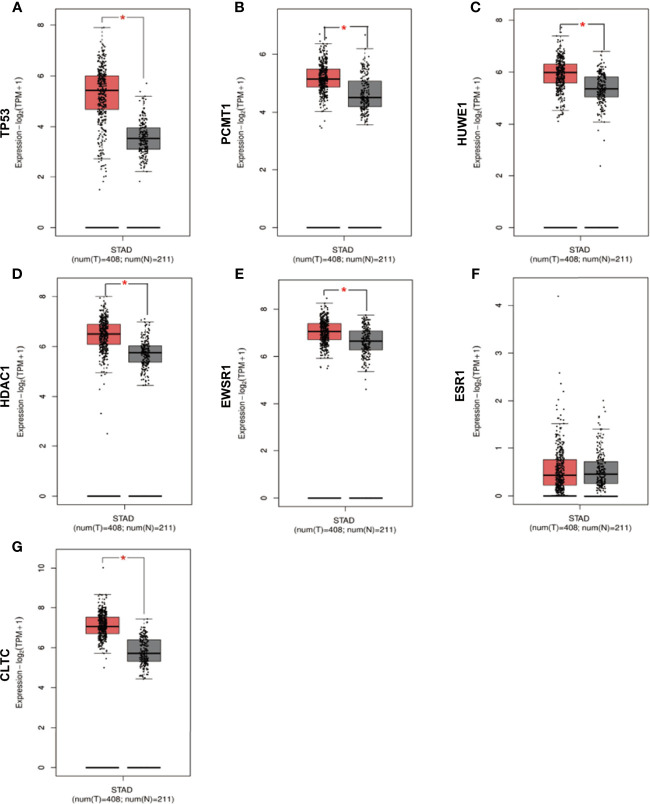
Verification of the expression level of 7 hub genes in STAD and normal tissues from GEPIA2 database. **(A)** Gene expression valueTP53 among samples of TCGA. **(B)** Gene expression value PCMT1 among samples of TCGA. **(C)** Gene expression value HUWE1 among samples of TCGA. **(D)** Gene expression value HDAC1 among samples of TCGA. **(E)** Gene expression value EWSR1among samples of TCGA. **(F)** Gene expression value ESR1 among samples of TCGA. **(G)** Gene expression value CLTC among samples of TCGA. Data are expressed in mean ± SEM; *P < 0.05, normal group versus tumor group.

**Figure 8:**
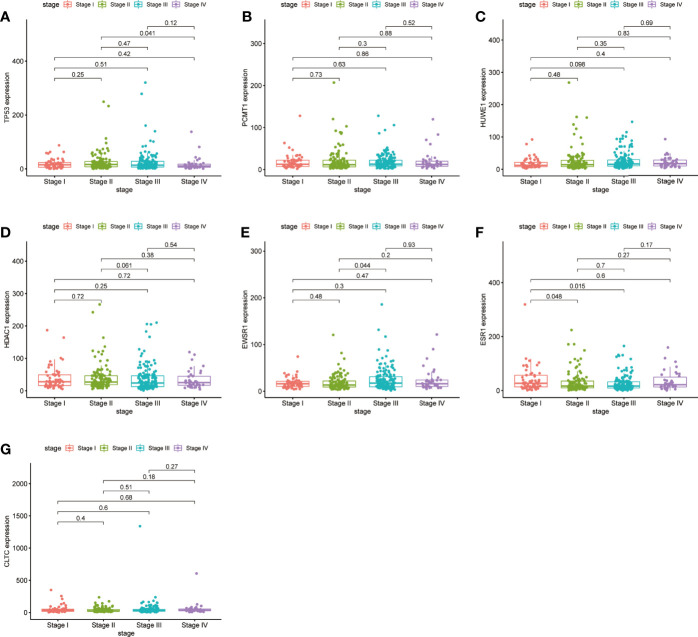
Validation of the expression levels of the seven hub genes of TCGA tissues from the TCGA database. **(A)** Gene expression value of *TP53* among samples of TCGA. **(B)** Gene expression value of *PCMT1* among samples of TCGA. **(C)** Gene expression value of *HUWE1* among samples of TCGA. **(D)** Gene expression value of *HDAC1* among samples of TCGA. **(E)** Gene expression value of *EWSR1* among samples of TCGA. **(F)** Gene expression value of *ESR1* among samples of TCGA. **(G)** Gene expression value of *CLTC* among samples of TCGA. *P < *0.05 was considered to be a statistically significant difference.

We checked the transformation level of the hub gene in diverse methods. As shown in **Figure 7**, the GEPIA2 database was used for analyzing the discrepant expression of seven hub genes in the normal group and the tumor group. In comparison with normal tissues, all hub genes were upregulated in stomach adenocarcinoma. For the follow-up exploration of the relationship between the seven hub genes and tumor stages, a block diagram was further drawn. *TP53* was expressed differently in stage 2 and stage 4 tumors, *EWSR1* was expressed differently in stage 2 and stage 3 tumors, and *ESR1* was expressed in stage 1 tumors. There were differences in the expression between the second and third phases (**Figure 8**).

**Figure 9:**
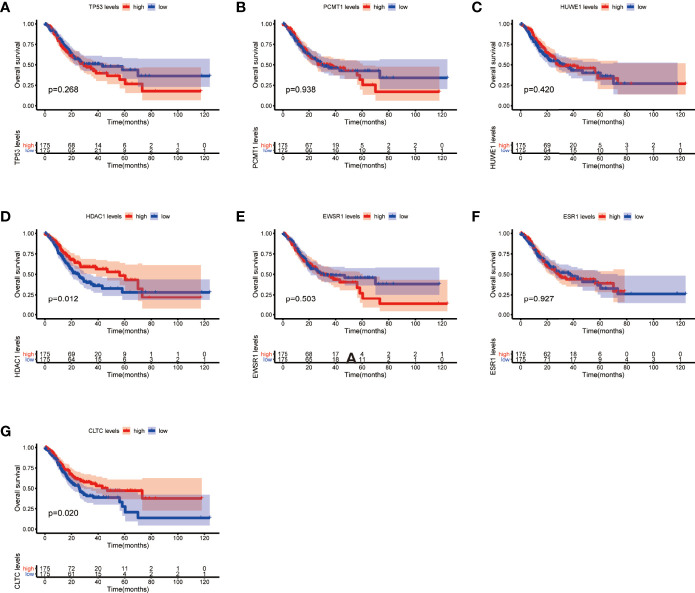
Overall survival (OS) analysis of the seven hub genes in STAD patients from the TCGA database. **(A)** Survival analysis for *TP53* in STAD. **(B)** Survival analysis for *PCMT1* in STAD. **(C)** Survival analysis for *HUWE1* in STAD. **(D)** Survival analysis for *HDAC1* in STAD. **(E)** Survival analysis for *EWSR1* in STAD. **(F)** Survival analysis for *ESR1* in STAD. **(G)** Survival analysis for *CLTC* in STAD. The patients were stratified into a high-level group (red) and a low-level group (blue) according to the median expression of the gene. Log-rank *P < *0.05 was considered to be a statistically significant difference.

**Figure 10:**
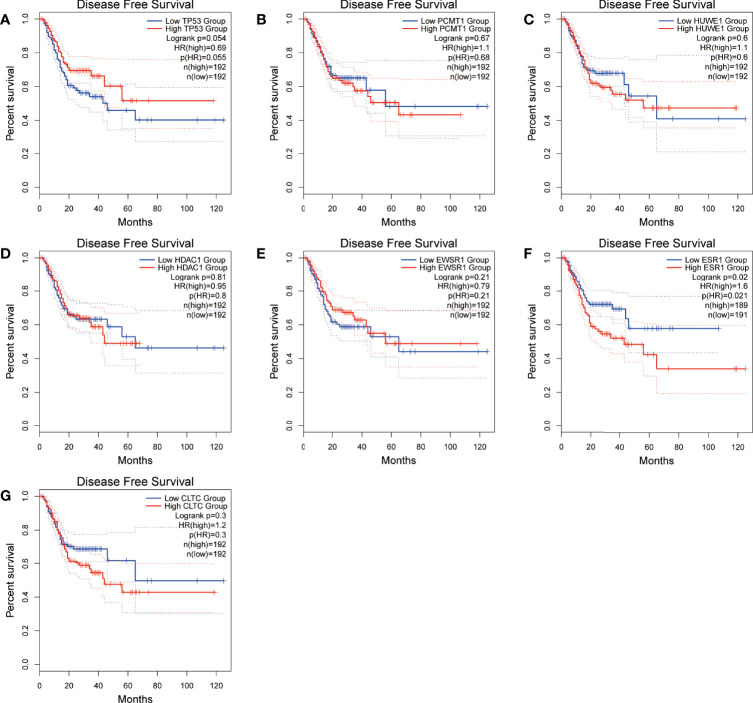
Disease-free survival (DFS) analysis of the seven hub genes in STAD patients from the GEPIA2 database. **(A)** Survival analysis for *TP53* in STAD. **(B)** Survival analysis for *PCMT1* in STAD. **(C)** Survival analysis for *HUWE1* in STAD. **(D)** Survival analysis for *HDAC1* in STAD. **(E)** Survival analysis for *EWS1* in STAD. **(F)** Survival analysis for *ESR1* in STAD. **(G)** Survival analysis for *CLTC* in STAD. The patients were stratified into a high-level group (red) and a low-level group (blue) according to the median expression of the gene. Log-rank *P < *0.05 was considered to be a statistically significant difference.

**Figure 11:**
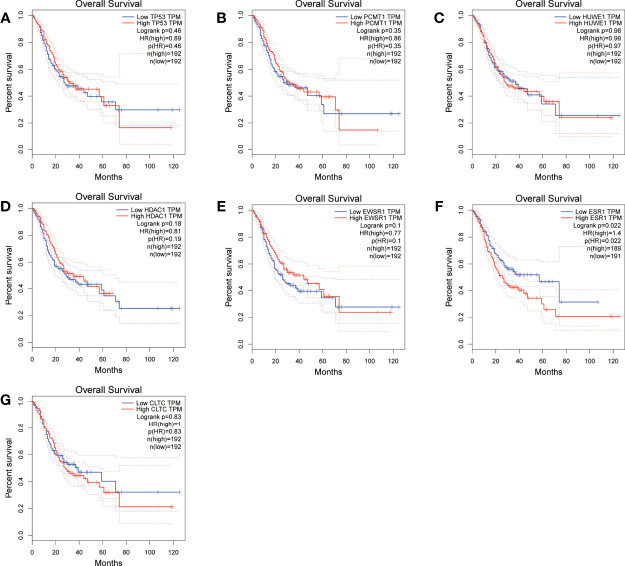
Overall survival (OS) analysis of the seven hub genes in STAD patients from the GEPIA2 database. **(A)** Survival analysis for *TP53* in STAD. **(B)** Survival analysis for *PCMT1* in STAD. **(C)** Survival analysis for *HUWE1* in STAD. **(D)** Survival analysis for *HDAC1* in STAD. **(E)** Survival analysis for *EWSR1* in STAD. **(F)** Survival analysis for *ESR1* in STAD. **(G)** Survival analysis for *CLTC* in STAD. The patients were stratified into a high-level group (red) and a low-level group (blue) according to the median expression of the gene. Log-rank *P < *0.05 was considered to be a statistically significant difference.

Moreover, we performed OS and DFS analysis on the seven hub genes using the R survival package and Kaplan–Meier plotter and the GEPIA2 database to study the clinical prognostic value of the hub genes in STAD patients. Among the seven hub genes, the Kaplan–Meier analysis of TCGA data showed that the expression levels of *HDAC1* and *CLTC* were drastically relative to the OS in stomach adenocarcinoma patients (*P* < 0.05) (**Figure 9**). GEPIA2 analysis showed that the expression of *ESR1* was correlated with the OS of stomach adenocarcinoma (**Figure 10**). It was closely correlated with DFS (*P* < 0.05) (**Figure 11**).

**Figure 12:**
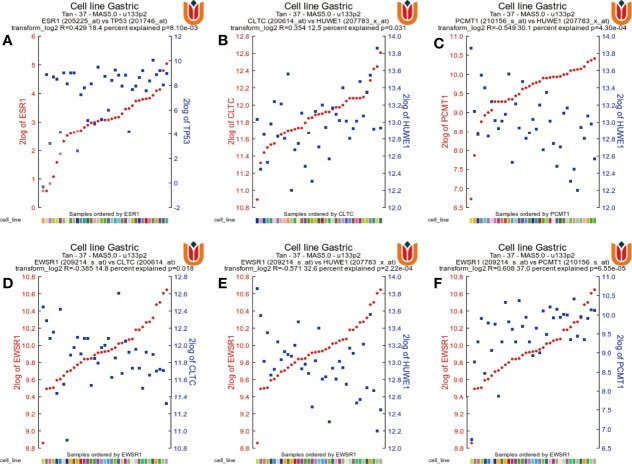
The gene correlations were revealed by the R2 Platform. **(A)** Correlation between the *ESR1* gene and the *TP53* gene (*R* = 0.429, *P* = 8.10e−03). **(B)** Correlation between *CLTC* and *HUWE1* (*R* = 0.354, *P* = 0.031). **(C)** Correlation between *PCMT1* and *HUWE1* (*R* = 0.549, *P* = 4.30e−04). **(D)** Correlation between *EWSR1* and *CLTC* (*R* = 0.385, *P* = 0.018). **(E)** Correlation between *EWSR1* and *HUWE1* (*R* = 0.571, *P* = 2.22e−04). **(F)** Correlation between *EWSR1* and *PCMTA* (*R* = 0.608, *P* = 6.55e−05).

Finally, to validate the expression correlation between the seven hub genes in STAD, the database was used and analyzed *via* the R2: Genomics Analysis and Visualization Platform (http://r2.amc.nl). As shown in **Figure 12**, *ESR1* expression was positively associated with *TP53* expression in STAD (*R* = 0.429, *P* = 8.10e−03). In addition, gene pairs that were related to each other also include *PCMT1* and *HUWE1*, *CLTC* and *HUWE1*, *EWSR1* and *HUWE1*, ESWR1 and *PCMT1*, and *EWSR1* and *CLTC*, which were all positively correlated.

**Figure 13:**
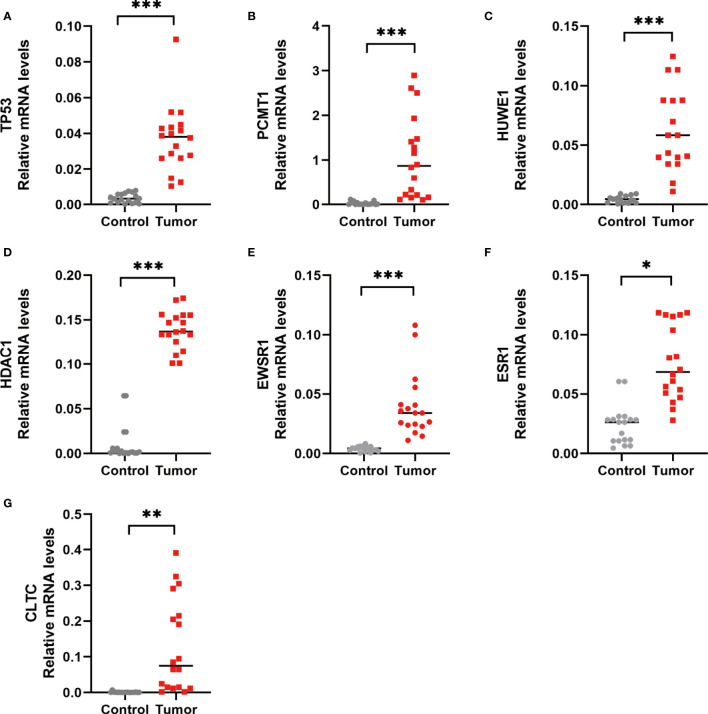
RT-PCR detection of the hub gene in normal and gastric adenocarcinoma tissues. **(A)** *TP53* expression level. **(B)** *PCMT1* expression level. **(C)** *HUWE1* expression level. **(D)** *HDAC1* expression level. **(E)** *EWSR1* expression level. **(F)** *ESR1* expression level. **(G)** *CLTC* expression level. *P < 0.05, **P < 0.01, and ***P < 0.001, control versus tumor, were considered to be statistically significant differences.

**Figure 14:**
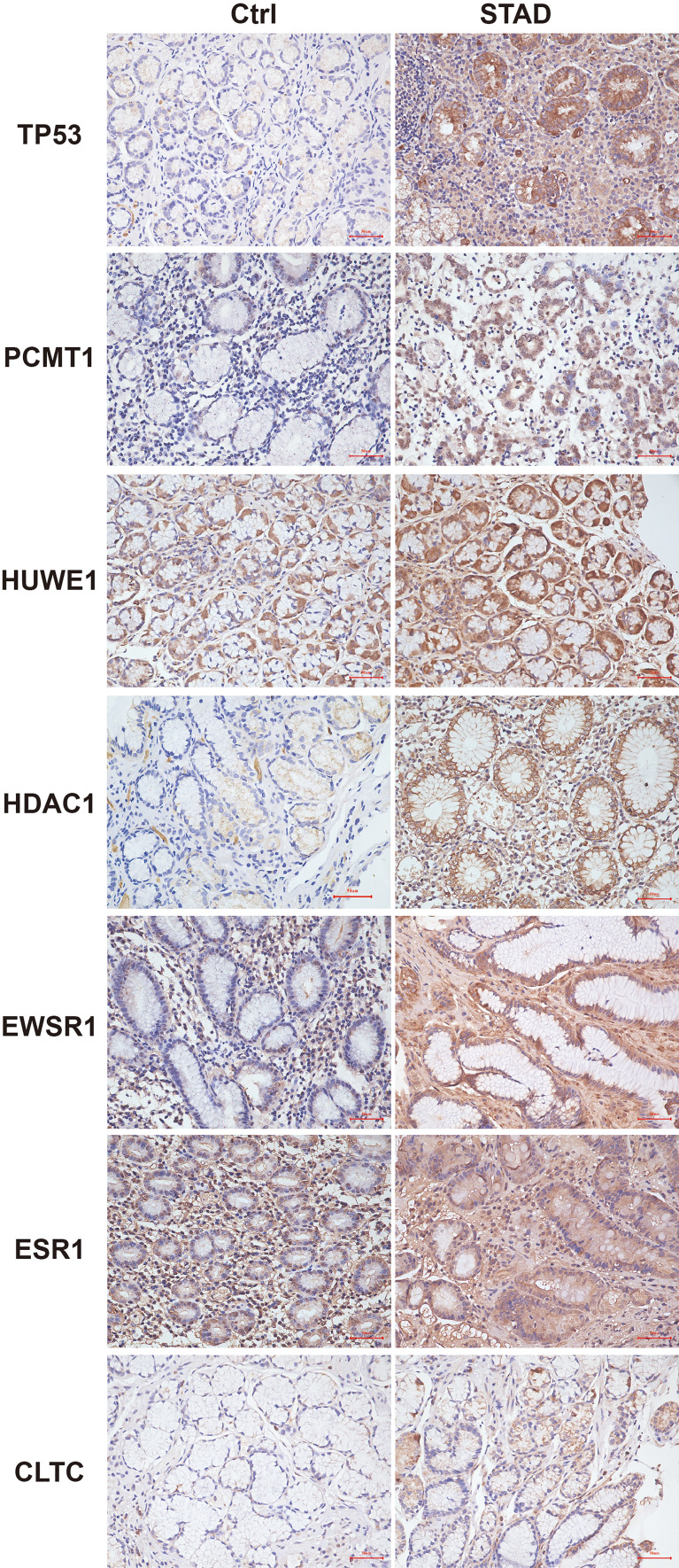
Immunohistochemistry of the seven hub genes in STAD and normal tissues. Brown indicates the intensity of the expressed protein.

Furthermore, to verify the outcomes of the bioinformatics analysis, we conducted RT-PCR and immunohistochemical staining on gastric adenocarcinoma tissues and normal tissues collected from the hospital. The outcomes showed that the expression level of the hub gene in tumor tissues was highly improved. This was consistent with the related protein expression levels as indicated by the immunohistochemical results (**Figures 13**, **14**).

## Discussion

Although there are various treatments for STAD, such as surgical resection, endoscopic resection, adjuvant chemotherapy, and immunotherapy (8), the prognosis of patients with advanced stomach adenocarcinoma is still very poor, and patients need to bear a lot of treatment costs. In recent years, target gene therapy has become a new treatment method, and some experiments have verified its effectiveness (9, 10), but the molecular mechanism of the pathophysiology of stomach adenocarcinoma is still unclear. Therefore, it is necessary to explore the susceptibility modules and genes of stomach adenocarcinoma and to further search for new biomarkers to predict the prognosis of stomach adenocarcinoma.

In this study, 42 core genes with the same expression trend were screened in the TCGA and GEO databases by integrating bioinformatics analysis. Analysis of the functional annotations of the clusterProfiler package shows that the results indicate a major focus on digestive processes, tissue homeostasis, hormone metabolic process, and maintenance of gastrointestinal epithelium enrichment. In the analysis of the signal pathway, it mainly involves stomach acid secretion, chemical carcinogenesis, collecting duct acid secretion, and other signal pathways. In addition, we established a PPI network and identified seven hub genes associated with STAD through Cytoscape’s cytoHubba plug-in. Compared with normal tissues, hub genes were upregulated in stomach adenocarcinoma (*P* < 0.05). Similarly, in our verification experiment, the expression of the hub genes showed the same trend. In addition, we found that the expression of *ESR1*, *HDAC1*, and *CLTC* genes in patients with stomach adenocarcinoma may be related to poor prognosis and lower overall survival.

EWSR1 is a polyfunctional protein that regulates cell function and aging by a variety of pathways, and is associated with the occurrence of mesenchymal tumors, multiple myeloma, Ewing’s sarcoma, and other tumors (11–13). PCMT1 is an unfavorable prognostic biomarker that participates in cell migration and invasion by regulating EMT-related genes (14). In our study, EWSR1 was shown to be differentially expressed in stages II and III of gastric cancer. There are no studies on EWSR1 and PCMT1 to confirm that they are related to the occurrence or prognosis of stomach adenocarcinoma, which can be used as points for further research.

HUWE1 encodes a protein containing a C-terminal HECT domain that acts as an E3 ubiquitin ligase. The protein was required for the ubiquitination and subsequent degradation of the anti-apoptotic protein Mcl1. It also ubiquitinates the p53, core histones, and DNA polymerase β. The latest research reported that HUWE1 can promote the proliferation, migration, and invasion of stomach cancer cells by mediating the ubiquitination of TGFBR2 (15). By comparing the expression level of HUWE1 in clinical gastric cancer patients and normal individuals, we showed that the expression level of HUWE1 was increased in tumor tissues. HUWE1 may become a potential target for the treatment of stomach cancer.

p53 is one of the most important tumor suppressors, involved in the regulation of a variety of tumor-related pathways, such as cell cycle, apoptosis, DNA damage repair, metabolism, inflammation and immune response, angiogenesis, and metastasis (16). Its coding gene *TP53* is the most common mutation gene in human cancer, while *TP53* mutation is usually associated with a poor prognosis of cancer (17). Related studies have shown that *TP53* mutations usually inhibit the body’s antitumor immunity and response to cancer immunotherapy (18–20). Conversely, studies have also reported that *TP53* mutations can promote antitumor immune activity and responsiveness to immunotherapy (21–23). These contradictory findings suggest that the correlation between *TP53* mutations and tumor immunity may be related to the type of cancer. Mutations in *TP53* are common and one of the five most important mutations in gastric cancer (24, 25). *TP53* has been proven to play an important role in the occurrence and development of gastric cancer (26, 27). Research confirmed that *TP53* affects the innate immune system by regulating macrophage function (28). *TP53* mutation also plays an important role in activating tumor immunity, which may promote the development of gastric cancer by affecting the immune characteristics of patients (18, 29). When *TP53* is mutated, cells proliferate abnormally and transform into cancer cells, and gastric cancer patients with *TP53* mutations have a worse prognosis than those without the mutation (30). *TP53* can be used to predict the prognosis of gastric cancer. Monitoring the recurrence of gastric cancer by monitoring free DNA mutations can also be used to predict the efficacy of chemotherapy in advanced gastric cancer (31, 32). Our study showed that compared with normal tissues, the expression of the *TP53* gene was upregulated in gastric adenocarcinoma, and there was a difference in stages II and IV. Our study showed that the expression of the *TP53* gene was upregulated in gastric adenocarcinoma compared with normal tissues, especially the difference in the expression between tumor stages II and IV.

A large number of studies have confirmed that *ESR1* can be used as a transcription factor to regulate many complex physiological processes of the human body and plays an important role in the treatment of many cancers, such as breast cancer, prostate cancer, and endometrial cancer (33, 34). *ESR1* has the function of encoding estrogen receptor and has been the focus of breast cancer research for a long time, but it is also related to gastric cancer and other types of cancer (35). Some research showed that activation of *ESR1* promotes the growth of breast cancer by triggering downstream signaling pathways, such as MAPK and PI3K (36). *ESR1* plays an important role in the occurrence and development of breast cancer. It can be used not only as a prognostic index but also as a predictor of endocrine therapy response (37, 38). In addition, *ESR1* is an oncogene that promotes the proliferation and metastasis of prostate cancer, and its expression is related to the poor prognosis of patients with prostate cancer (39). There is increasing evidence that estrogen affects the proliferation and tumor progression of the prostate epithelium through the *ESR1* signal (40). Although the stomach is not the direct target organ of estrogen, in recent years, accumulating clinical and laboratory evidence has shown that estrogen is closely related to gastric cancer, but its specific mechanism is still unclear to a large extent (41, 42). Estrogen and ESR play not only a normal physiological function in regulating body growth and development but also an important role in the growth, proliferation, invasion, and metastasis of gastric cancer (43, 44). Our results showed that the level of *ESR1* was increased in gastric adenocarcinoma patients. We speculate that *ESR1* has diagnostic value in clinicopathological features and prognosis of patients with gastric cancer, and selective targeting of the estrogen receptor may be a new therapeutic tool to eliminate tumor growth and metastasis.

*HDAC1* is a protein-coding gene, which can catalyze histone deacetylation reaction, and downregulates histone acetylation level, which can compress chromatin into a dense conformation and decrease transcriptional activity (45). Studies have confirmed that *HDAC1/2* is significantly increased in many human cancers (46–48), especially in the invasiveness and carcinogenicity of gastric cancer (49, 50). Its high expression is related to advanced stomach cancer, uncontrolled tumor cell proliferation, and poor prognosis (51). The expression of *HDAC1* is also one of the independent poor prognostic factors for the overall survival and disease-free survival of patients with stomach cancer (52). Some studies have further shown that the high expression of HDACs is clinically related to lymph node spread and shorter overall survival time in patients with gastric cancer (51, 53). Our results also confirmed that *HDAC1* was correlated with tumor occurrence, development, and clinical prognosis. Therefore, *HDAC1/2* is an effective therapeutic target for STAD. It has been confirmed that the HDAC inhibitor trichostatin A plays an antiproliferation effect by regulating cell cycle and apoptosis and can increase the chemical sensitivity of gastric cancer cell lines to anticancer drugs, including 5-fluorouracil, PTX, and irinotecan (54, 55). It is of great significance to further develop more alternative treatment strategies in the future and to improve the treatment of patients with gastric cancer.

Clathrin is a protein that plays a major role in the formation of coated vesicles. It forms a triangular shape composed of three clathrin heavy chains and three light chains that spontaneously assemble into a basket lattice to drive the budding process of endocytosis (56). Clathrin heavy chain (CLTC) plays an important role in the uptake of exosomes by the mononuclear phagocyte system (MPS). It has been found that MPS endocytosis in the spleen and liver can be significantly blocked by pre-injection of exosomes loaded with siRNA against CLTC, which then leads to subsequent increased delivery of exosomes in other organs (57). Further studies also confirmed that the blocking strategy using siCLTC-modified exosomes could significantly improve the protective effect of specific exosomes in a model of doxorubicin-induced cardiotoxicity (57). In cancer research, CLTC has also been reported to be involved in tumorigenesis. The *CLTC*–*ALK* fusion gene has been shown to be an ALK activator in large B-cell lymphoma and is associated with tumor recurrence (58). This abnormal fusion gene is also thought to be a major factor in congenital primitive plasmacytoid dendritic cell tumors (59). The high expression of CLTC has also been shown to be an independent prognostic factor for tumor-free survival and overall survival in patients with osteosarcoma (60). Transcriptome analysis revealed that CLTC–TFE3 fusion is present in renal cancer and affects many downstream cancer-related pathways (61). A great deal of evidence supports the concept that CLTC fusion protein is involved in tumorigenesis and tumor progression. However, the role of CLTC in gastric adenocarcinoma has not been further studied. Our results show that the expression of CLTC is increased in gastric cancer patients, which is of great significance for the treatment of gastric adenocarcinoma and further exploration of CLTC.

Among the seven hub genes screened, *TP53* is the most frequently mutated gene in various human cancers, and 90% of *TP53* mutations are missense changes with potential gain-of-function features (62). Cheng et al. highlight the important role of *TP53* genomic status in influencing gastric cancer response to DZNep (3-deazacycline A). When evaluating clinical trials of EZH2-targeted agents, such as DZNep, consideration should be given to stratifying gastric cancer patients according to their *TP53* genomic status (24). The other six hub genes have no gene mutation records in STAD-related research, but *HDAC1* and *ESR1* genes have been confirmed to be related to the occurrence and prognosis of gastric adenocarcinoma, associated with the patients’ DFS and OS. The remaining genes have not been studied in the pathogenesis and treatment of STAD. However, we analyzed clinical patient samples and found that the expression of those genes was elevated.

Secondly, the relationships between these hub genes were further studied. In view of the relatively few related literature, we have found only a few pairs of genes that may be associated, including *HDAC1* and *ESR1*, *HDAC1* and *TP53*, and *CLTC* and *TP53*, as well as *TP53* and *ESR1*. *HDAC1* and *ESR1* genes play an important role in regulating the Notch signal transduction pathway (63). Premature ovarian failure (POF) can also be treated by regulating the balance of *ESR* in the *TP53*–*AKT* signaling pathway (64). Studies have shown that loss of heterozygosity (LOH) of *TP53* and *ESR* is higher in ovarian serous cystadenocarcinoma (SCA) and more common in clear cell carcinoma (CCA) and serous tumor with low malignant potential (SLMP), which has been observed in primary ovarian tumors and metastases (65). *CLTC*–*VMP1* gene fusion and *TP53* gene mutation were observed in undifferentiated pleomorphic osteosarcoma (66). The rearrangement of the RNA-binding protein EWSR1 characterizes a variety of malignant tumors, including Ewing’s sarcoma (EWSR1/ETS), depilated small round cell tumor (EWSR1/WT1), and some acute lymphoblastic leukemia (EWSR1/ZNF384) (67). Although these fusions involve known cancer genes, they all occur in new fusion partners and previously unreported types of cancer. Some repeatedly mutated genes and gene fusion represent potential drug targets, which can be transformed into a diagnostic basis and can be used in clinical treatment and improvement in the prognosis of patients.

## Conclusion

Due to the clinical heterogeneity of patients and the small number of included samples, the number of relevant references that can be retrieved is also small, so there are some limitations. Although we conducted a systematic bioinformatics analysis and identified the potential differential hub genes between tumor and normal tissues, the correlation between the expression of hub genes and its clinical significance is difficult to determine. Moreover, further examinations are needed to clarify their prognostic information. However, the seven hub genes are still expected to be used as potential STAD molecular biomarkers in the future. Further study of these genes can broaden our understanding of the pathogenesis of STAD and highlight the possibility of developing new targeted therapeutic drugs.

## Data Availability Statement

The original contributions presented in the study are included in the article/**Supplementary Material**. Further inquiries can be directed to the corresponding author.

## Ethics Statement

The studies involving human participants were reviewed and approved by the Shanghai Municipal Hospital of Traditional Chinese Medicine. The patients/participants provided their written informed consent to participate in this study.

## Author Contributions

KZ performed the data acquisition and analysis and wrote the manuscript. JW participated in the revision of the article and data query. The other authors conducted the experimental operation and data collection and analysis. YL conceived the study design and is the guarantor of the article. All authors contributed to the article and approved the submitted version.

## Funding

This work was supported by the National Natural Science Foundation of China (No. 81573775; No. 81873157) and the Postgraduate Innovation Training Special Project (Y2020020).

## Conflict of Interest

The authors declare that the research was conducted in the absence of any commercial or financial relationships that could be construed as a potential conflict of interest.

## Publisher’s Note

All claims expressed in this article are solely those of the authors and do not necessarily represent those of their affiliated organizations, or those of the publisher, the editors and the reviewers. Any product that may be evaluated in this article, or claim that may be made by its manufacturer, is not guaranteed or endorsed by the publisher.

## Supplementary Material

Supplementary Table 1 Information of patients.
