# Supplementary material for: Identification of Hub Genes Associated With the Development of Stomach Adenocarcinoma by Integrated Bioinformatics Analysis
Source: Front Oncol. 2022 May 24;12:844990. doi: 10.3389/fonc.2022.844990 (PMC9170954; doi:10.3389/fonc.2022.844990)
Supplement: Supplementary Table 1 — Information of patients. [file Table_1.docx]

**Supplementary Table**

**Table. Informations of Patients**
